# Breakthroughs in Oral and Maxillofacial Surgery

**DOI:** 10.3390/jcm13030685

**Published:** 2024-01-24

**Authors:** Alessandro Antonelli, Francesco Bennardo, Amerigo Giudice

**Affiliations:** School of Dentistry, Department of Health Sciences, Magna Graecia University of Catanzaro, 88100 Catanzaro, Italy; francesco.bennardo@unicz.it (F.B.); a.giudice@unicz.it (A.G.)

## 1. Introduction

In the field of oral and maxillofacial surgery, continuous advances have ushered in a new era of innovation, profoundly influencing this branch of medicine. Cutting-edge technologies, techniques, and detailed analysis of the patient’s local and general clinical conditions have transformed traditional surgical procedures, improving precision, efficiency, and patient outcomes [1,2].

From individual case planning using surgical simulation software to the use of 3D printing to create stereolithographic models and customized prostheses, the landscape of oral surgery has evolved significantly [3].

## 2. Discussion

Breakthroughs in oral and maxillofacial surgery have been driven by continuous research, innovation, and collaboration among surgeons, researchers, and engineers. The development and improvement in surgical techniques have enabled complex procedures such as orthognathic surgery, oncologic, and reconstructive surgery and the management of facial trauma to be performed with greater precision and improved outcomes [4,5,6]. Furthermore, the introduction of advanced imaging techniques, such as computed tomography (CT) scans and cone beam computed tomography (CBCT), has revolutionized diagnosis and treatment planning by providing detailed 3D images of the oral and facial structures [7,8,9].

In addition, the integration of three-dimensional imaging for the analysis of bone structures with non-invasive devices that enable the analysis of facial soft tissue has made it possible to more accurately assess the benefits that patients can derive from new surgical methods and custom-made treatment plans [10].

This background includes the latest generation of facial scanners and devices accessible to everyone, such as smartphones, that allow the capture of high-resolution 3D images of the patient’s facial anatomy with remarkable precision [11]. Surgeons leverage this technology to create detailed digital models, enabling them to analyze complex craniofacial structures and plan interventions with unprecedented accuracy [12,13]. In fact, the relevance of encoded parameters becomes even more pronounced in the field of precision medicine, where tailored treatment strategies are designed based on individualized patient profiles [14].

As a result of continuous innovations in the medical and pharmacological fields, researchers have also had to find solutions to possible adverse reactions due to interactions with new drugs [15,16]. In particular, the development of new monoclonal antibodies for the management and treatment of oncological pathologies has had important repercussions on the management of pathologies associated with the action of these drugs on the maxillary bone [17].

The importance of the management of these patients, often subjected to multiple therapies, has highlighted the relevance of the management of possible odontogenic infections that can trigger adverse processes such as medication-related osteonecrosis of the jaws [18]. In fact, researchers have recently demonstrated that the focus of this pathology is the inflammatory-infective state of the periodontal and alveolar bone, which is the main clue for the onset of this adverse drug reaction [18,19].

In recent years, during the COVID-19 pandemic, significant advancements and breakthroughs in oral surgery have emerged, driven by the need for enhanced safety measures and improved patient care [20]. The usefulness of telemedicine and virtual consultations has streamlined the pre-operative assessment process, reducing hospital access and minimizing potential exposure to the virus [20,21]. Furthermore, the rigorous infection control protocols implemented in oral surgery units have not only safeguarded patients and healthcare providers but have also set new standards for hygiene and safety in the field [22,23,24].

Another field of oral and maxillofacial surgery in which researchers have developed new procedures and analyzed biological phenomena is prosthetic implant surgery [25]. Although great emphasis has been placed on guided bone regeneration (GBR) procedures over the years, they are still often unpredictable and difficult to perform [26]. For this reason, clinicians and researchers have developed implant designs in consultation with engineers to exploit as much residual native bone as possible [27,28]. Equally, interest has been placed on new surgical methods for implant site preparation to modify the bone density at the osteotomy site walls to achieve higher and more predictable primary stability values [29,30,31,32,33,34]. In fact, it has been observed that a parameter such as primary implant stability is directly linked to the biological phenomenon of osseointegration and thus obtains a better success rate of implant prosthetic therapy in the medium to long term [35,36]. Precisely for this reason, another biological factor that is monitored over time is crestal marginal bone loss, which can occur over time and lead to possible peri-implantitis phenomena [37,38,39].

Taken together, the research included in this Special Issue highlights the relevance of new discoveries and breakthroughs in oral and maxillofacial surgery to improve routine care for a wide range of diseases that impact patient health. Furthermore, it is necessary to consider how a patient-specific approach has become necessary for optimal management of each individual clinical scenario.

## 3. Conclusions

In light of these considerations, clinicians and researchers should be aware of the need to implement notions coming from the medical and surgical fields with modern technological systems that benefit from engineering concepts for new therapeutic approaches that are changing the clinical scenario of facial pathologies. The relevance of breakthroughs in oral and maxillofacial surgery has become even more evident in the field of precision medicine, where tailored treatment strategies are designed based on individualized patient profiles.
